# Infodemic Signal Detection During the COVID-19 Pandemic: Development of a Methodology for Identifying Potential Information Voids in Online Conversations

**DOI:** 10.2196/30971

**Published:** 2021-07-28

**Authors:** Tina D Purnat, Paolo Vacca, Christine Czerniak, Sarah Ball, Stefano Burzo, Tim Zecchin, Amy Wright, Supriya Bezbaruah, Faizza Tanggol, Ève Dubé, Fabienne Labbé, Maude Dionne, Jaya Lamichhane, Avichal Mahajan, Sylvie Briand, Tim Nguyen

**Affiliations:** 1 Digital Health and Innovation Science Division World Health Organization Geneva Switzerland; 2 Media Measurement Ltd London United Kingdom; 3 Emergency Preparedness World Health Organization Geneva Switzerland; 4 Department of Political Science University of British Columbia Vancouver, BC Canada; 5 Health Emergencies Programme World Health Organization Regional Office for South East Asia New Delhi India; 6 World Health Organization Country Office Malaysia Brunei Darussalam and Singapore Putrajaya Malaysia; 7 Institut national de santé publique du Québec Montreal, QC Canada

**Keywords:** infodemic, COVID-19, infodemic management, social listening, social monitoring, social media, pandemic preparedness, pandemic response, risk communication, information voids, data deficits, information overload

## Abstract

**Background:**

The COVID-19 pandemic has been accompanied by an *infodemic*: excess information, including false or misleading information, in digital and physical environments during an acute public health event. This infodemic is leading to confusion and risk-taking behaviors that can be harmful to health, as well as to mistrust in health authorities and public health responses. The World Health Organization (WHO) is working to develop tools to provide an evidence-based response to the infodemic, enabling prioritization of health response activities.

**Objective:**

In this work, we aimed to develop a practical, structured approach to identify narratives in public online conversations on social media platforms where concerns or confusion exist or where narratives are gaining traction, thus providing actionable data to help the WHO prioritize its response efforts to address the COVID-19 infodemic.

**Methods:**

We developed a taxonomy to filter global public conversations in English and French related to COVID-19 on social media into 5 categories with 35 subcategories. The taxonomy and its implementation were validated for retrieval precision and recall, and they were reviewed and adapted as language about the pandemic in online conversations changed over time. The aggregated data for each subcategory were analyzed on a weekly basis by volume, velocity, and presence of questions to detect signals of information voids with potential for confusion or where mis- or disinformation may thrive. A human analyst reviewed and identified potential information voids and sources of confusion, and quantitative data were used to provide insights on emerging narratives, influencers, and public reactions to COVID-19–related topics.

**Results:**

A COVID-19 public health social listening taxonomy was developed, validated, and applied to filter relevant content for more focused analysis. A weekly analysis of public online conversations since March 23, 2020, enabled quantification of shifting interests in public health–related topics concerning the pandemic, and the analysis demonstrated recurring voids of verified health information. This approach therefore focuses on the detection of infodemic signals to generate actionable insights to rapidly inform decision-making for a more targeted and adaptive response, including risk communication.

**Conclusions:**

This approach has been successfully applied to identify and analyze infodemic signals, particularly information voids, to inform the COVID-19 pandemic response. More broadly, the results have demonstrated the importance of ongoing monitoring and analysis of public online conversations, as information voids frequently recur and narratives shift over time. The approach is being piloted in individual countries and WHO regions to generate localized insights and actions; meanwhile, a pilot of an artificial intelligence–based social listening platform is using this taxonomy to aggregate and compare online conversations across 20 countries. Beyond the COVID-19 pandemic, the taxonomy and methodology may be adapted for fast deployment in future public health events, and they could form the basis of a routine social listening program for health preparedness and response planning.

## Introduction

### Background

Since the beginning of the COVID-19 pandemic, digital communication and social networking have supported the rapid growth of real-time information sharing about the virus that causes COVID-19 (SARS-CoV-2) and the disease in the public domain and across borders. The breadth of conversation, diversity of sources, and polarity of opinions have sometimes resulted in excessive information, including false or misleading information, in digital and physical environments during an acute public health event; this can lead to confusion and risk-taking behaviors that can harm health, trust in health authorities, and the public health response [1]. The excess of information can amplify and protract outbreaks, and it can reduce the effectiveness of pandemic response efforts and interventions.

To address this challenge, the World Health Organization (WHO) Information Network for Epidemics (EPI-WIN), in collaboration with digital research partners, developed a methodology for weekly analysis of digital social media data to identify, categorize, and understand the key concerns expressed in online conversations [2]. The application of this methodology provided the WHO with week-on-week analysis for the prioritization of actions to address online information voids and sources of confusion using verified health information as part of ongoing emergency response planning. When there is a lack of quality information about topics of concern for online users, these topics can be quickly filled with conjecture, low-quality health information, and viral misleading content [3,4], thus potentially causing harm to communities. This approach therefore focuses on the detection of infodemic signals—identifying or predicting rising areas of concern and information voids in the online information ecosystem on a weekly basis to generate actionable insights to rapidly inform decision-making for a more effective response, including adapting risk communication [5].

### Infodemic Management During a Health Emergency

Previous research has explored the use of data produced and consumed on the web to inform public health officials, agencies, and policy—a concept known as *infodemiology* [6]. Initially, the concept of infodemiology aimed to identify the gap between expert knowledge and public practice [7], and it has since evolved to detect and analyze health information on the web through publicly shared search queries, blogs, websites, and social media posts. 

The design of interventions for infodemic response must account for an ecosystem where information flow online can cause public health harm offline. Metrics and frameworks related to digital information flows and online behavior are most useful to practitioners when they can be coupled with other online and offline sources of public health data that inform public health decision-making. The WHO has therefore expanded the concept of infodemiology into a multidisciplinary scientific field that amalgamates cross-disciplinary and mixed methods approaches designed to inform the health emergency response [8].

Health emergencies give rise to information overload, which has been shown to influence people’s risk perceptions and protective actions during health emergencies [9]. Overload of information of variable quality, timeliness, and relevance is strongly associated with people’s experience of information anxiety, which in turn can give rise to information avoidance. Recent examples, from HIV to Ebola virus to Zika virus to polio, have demonstrated the high cost to public health and health systems when misinformation sows distrust, exacerbated by ineffective public health communication and community engagement [3,10]. A lack of active community collaboration in the health response early on deepened distrust, especially as these epidemics unfolded. Currently, most emergency and outbreak recommendations emphasize the value of listening to communities, involving them early in the response, and communicating clearly with them in a timely manner [11,12].

Health authorities therefore not only face the challenge of providing relevant, high-quality health information but also must provide it at the right time, in the right format, and with collaborative engagement of communities [13]. Social listening can help overcome barriers to acceptance of high-quality health information and enactment of healthy behaviors by enabling better understanding of community questions, confusion, information seeking, or intensified attention for given topics. Critical information voids can be identified and characterized in both the online and offline information ecosystems. Our research focuses on the identification and characterization of points of confusion, harmful narratives, and key questions that can reveal information voids in the online social media space during a health emergency, thereby adding analytical methods to the field of infodemiology that are practical and can directly inform the public health response during a health emergency.

### Analytical Approaches and Metrics To Date

The rise of social media platforms has generated a readily available source of real-time data related to what people express and share in online communities. The 2009 H1N1 influenza pandemic was the first pandemic to occur in the era of social media and was one of the earliest outbreaks informed by analysis of online conversations and information-seeking behaviors. The previous pandemic offers a case study that evidences how online social listening has been used to follow rapidly evolving public sentiment, track actual disease activity, and monitor the emergence of misinformation [14-16]. Although social media platforms have been used to quantify public concerns and sentiment and to monitor real-time pandemic data, they have also been identified as a medium that can enable the spread of low-quality information. For example, within health emergencies, false information has been shown to be posted twice as frequently as evidence-based information, although it is retweeted less frequently [17]. Provision of targeted, relevant, timely, understandable, and resonant health information can therefore benefit from upstream infodemic management activities of public health authorities, including more robust social listening programs.

The onset of the COVID-19 pandemic has exacerbated concerns about misinformation. Throughout the pandemic, there has been a demand for information; at first, this demand was for information about the origin of the virus, and now it is focused mainly on the response to the virus, particularly vaccination and wider public health and social measures. Similar to information voids [5], COVID-19 misinformation trackers have defined the concept of *data deficits* in the online space when there are “high levels of demand for information about a topic, but credible information is in low supply” [3]. The issue with a lack of quality information is that the conversation space can be much more readily filled by misinformation, which may be faster to create and share, more emotive (resonant), and better promoted by content promotion algorithms than factual health information.

Despite the influx of studies as to how information is being spread and shared in the era of COVID-19 and how information is influencing people’s health practices, major gaps in knowledge remain as to how best to monitor, understand, and respond to it [8]. Among many possible solutions, social listening, content pretesting, and other computational social science methods have been identified as ways to detect and analyze information voids and viral misinformation narratives [13]. Misinformation research has focused on social media platforms with easier access to data, such as Twitter and YouTube [18,19]; however, misinformation is prevalent across the digital ecosystem (as well as offline). Culture and access to the internet can also affect the nature of misinformation and how it spreads [20]. Beyond identifying what misinformation looks like, studies have also attempted to identify how it emerges [21]*,* aligning with the concept of information voids. Although social listening has tended to focus on spotting myths and rumors, as well as content items with high engagement and reshare rates, the methodology introduced in this paper expands the scope of social listening and positions it as a core practice of emergency response. This includes prioritizing detection of information voids for more proactive infodemic management before these gaps in understanding are filled with more speculation, misleading information, and counterproductive narratives.

Detecting viral misinformation narratives and information voids in real-time data is crucial to a rapid, comprehensive response by authorities for effective delivery of health information to populations during a health emergency, although this does not ensure that people will necessarily act in accordance with that health information. Previous research has evaluated the correction of misinformation and the role of individuals versus organizations in using real-time data [22-24]. The pathway from receiving information, to intent, to action is understudied and a priority area for future research [8]. Evaluation of intervention impact is challenging [4], but evaluation of interventions must be integrated as part of adaptive infodemic management, including social listening.

Interventions need to address the different aspects of the information ecosystem that influence the spread and health impact of an infodemic. For platforms, content moderation policies, modification of content promotion algorithms, and designing for friction can discourage sharing of misinformation and unverified information [25], while supporting literacies such as health, media, information, digital, and data literacies can promote resilience [26]. The literature highlights the value of a multipronged approach for addressing infodemics at various levels in the digital information ecosystem. However, although public health authorities can influence and interact with the other participants in this space, there is a need to suggest immediate and practical tools that public health authorities can deploy within their mandate in a health emergency context in support of their health operations and communication activities [8].

### A Need for Practical Tools for Health Authorities

Research is ongoing to assist policy makers in understanding public concerns and sentiment around the pandemic as well as in tracking information outbreaks and the emergence of misinformation. However, there is little to no empirical evidence on how this research can be used to develop practical tools for an outbreak response by public health authorities. More collaborations between researchers and public health practitioners are needed to fill this gap. As a contribution to the infodemic response toolbox, the taxonomy and methodology in this study offer a practical, structured approach for identifying information voids and narratives of concern that warrant attention and action. This approach has already provided actionable data to help the WHO focus its efforts for the COVID-19 pandemic response.

## Methods

### Development of a Public Health Taxonomy for Social Listening

A social listening taxonomy for COVID-19 conversations was developed specifically for this analysis. It was designed to filter digital content referring to COVID-19 (and synonyms) for items of relevance in a public health context and to classify that content into categories. The taxonomy consisted of 35 keyword-based searches (one set of searches for each of two languages, namely English and French) which were grouped into 5 overarching topic categories representing thematic areas in which people were engaging, writing, or searching for information.

**Figure 1:**
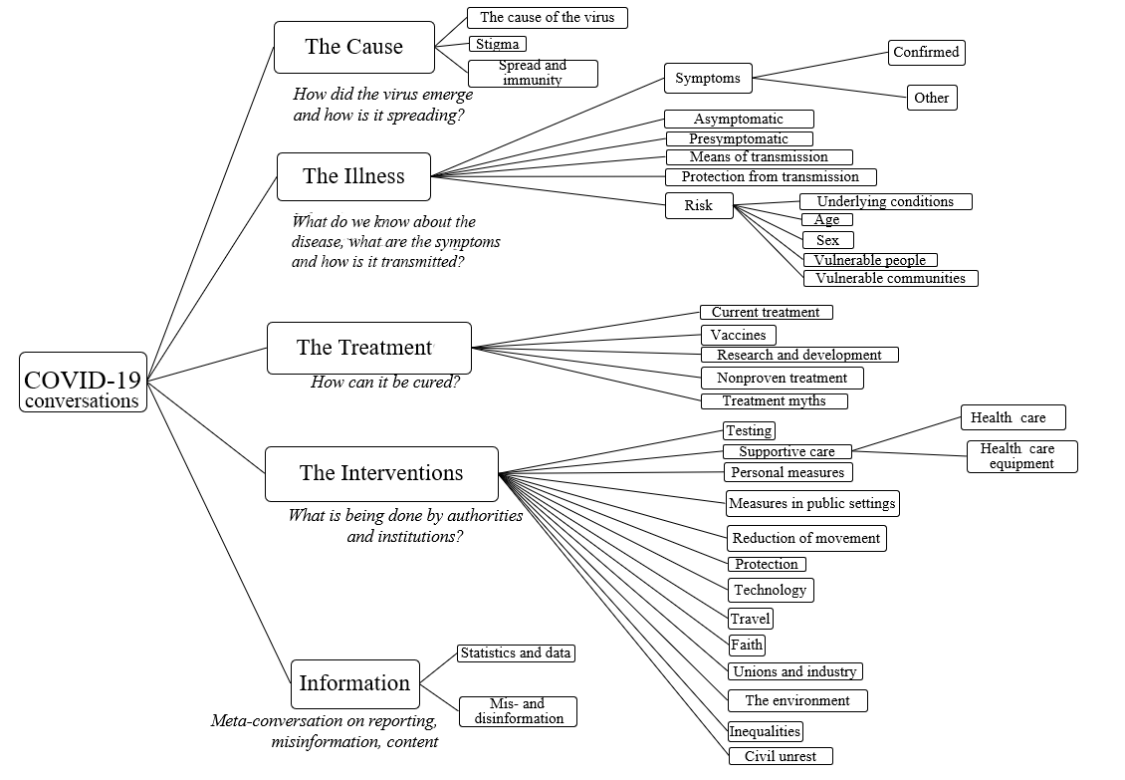
Structure of the social listening taxonomy for COVID-19 conversations.

The 5 top-level categories and corresponding 35 subcategories of this social listening taxonomy for COVID-19 conversations were defined based on established epidemic management and public health practices during an outbreak of infectious disease [27] (Figure 1). The first 4 categories refer to the focus of epidemic management activities during the pandemic: (1) the cause of the disease—what do we know about the virus, and how is it spreading? (2) the illness—what are the symptoms, and how is it transmitted? (3) the treatment—how can it be cured? and (4) the interventions—what is being done by authorities and institutions? In addition, a fifth category was included to examine public perceptions on circulating information (ie, metaconversations about evidence and statistics, mis- and disinformation, successful and harmful content, or key influencers who have been actively amplifying information on COVID-19). This category was designed because misinformation, rumors, and polarization of factual versus misleading narratives are common challenges in epidemic management.

Each of these 5 categories were segmented into subcategory levels that are familiar to the epidemiologist’s investigation and management of the outbreak, resulting in a total of 35 taxonomy subcategory levels (Figure 1). For example, the taxonomy category about the illness was further defined by subcategories to identify conversations, questions, or confusion about the symptoms of the illness, how it transmits, and what populations may be affected by it (across demographics, vulnerable populations, and people with underlying conditions). By defining the social listening taxonomy across the investigation areas of epidemic management [27], the resulting infodemic insights can be more quickly evaluated by public health professionals and turned into actionable recommendations to inform the epidemic response.

Each of the 35 taxonomy subcategories encompassed a list of topics that captured different aspects of that segment of the online conversation on COVID-19. Keywords for the 35 subcategory searches were generated based on expert knowledge from the WHO EPI-WIN team and translated into Boolean search strings to identify topic-related language for review of relevant social media posts and news content. The keywords of the taxonomy are available on request at the contact address listed in the *Acknowledgments* section.

In addition to the taxonomy subcategory levels, the keyword-based Boolean search string was created to also identify posts containing a question; this enabled analysis of categories for which people were seeking information and, therefore, potential information voids. The question search string was designed to be paired with each of the 35 taxonomy Boolean search strings to identify posts referring to the topic and containing question words, verb-subject inversions, and auxiliaries.

Finally, the sum of the total volume of the social media conversation (on all topics) was estimated by monitoring the number of posts mentioning at least one of the most commonly used words in English (eg, *the*, *and*, *or*, *I*) and French (eg, *le*, *la*, *ou*, *et*). The data were collected via a Boolean search string comprised of these most commonly used words.

### Data Sources and Data Collection

The analysis was based on the weekly aggregation of publicly available social media data in English and French using Meltwater Explore. Institutional Review Board review was not sought, as the analysis used large bodies of text written by humans on the internet and on some social media platforms. The analysis and resulting reports focused on the identification of conversation narratives and thematic questions instead of on individual statements and users.

The Meltwater social listening platform was configured to collect verbatim mentions of keywords associated with the 35 predefined taxonomy category Boolean searches from 9 open data sources and fora (Twitter, blog entries, Facebook, Reddit posts and comments, other unspecified message boards or fora, comments under news articles and blog entries, Instagram posts, product reviews, and YouTube video titles and comments). A total of 87.02% of the resulting analysis data set was sourced from Twitter. Blogs (5.34%) and, specifically, the Reddit platform (4.34%) were the next most prominent sources in the data set. These were followed by message boards (2.14%), comments under news articles (0.89%), online review websites (0.13%), Instagram (0.12%), and Facebook (0.03%).

For each of the 35 taxonomy subcategories, the global daily total volume of posts, and the volume of posts posing a question, were recorded on a weekly basis. Tracking changes in volume from week to week also enabled determination of the velocity for a given subcategory.

### Testing and Validation of the Taxonomy

The methodology used to test and validate the retrieval and classification in this study used both retrieval precision and retrieval recall, which are related to how much retrieved data is relevant and how much relevant data is retrieved, respectively [28,29]. These validation metrics are useful for assessing the performance of machine learning models in information retrieval and have been used for metrics on content retrieved and classified via Boolean searches for news media content [28] and Twitter data [29].

To test whether the taxonomy categories captured the intended information (retrieval precision), a random sample of content captured by each of the 35 Boolean searches was human-coded for relevance (10,500 posts in total) by a single reviewer, with a second reviewer validating the coding. The post was coded as either relevant to the search subcategory (1) or not relevant (0).

The aim of the coding was to determine the proportion of relevant (R; also, “true positive” [TP]) results as a percentage of the retrieved sample. The coders judged whether a post was relevant according to the intended definition of the specific subcategory search for which the post was returned. For example, if a post had been returned for the “The Illness – Confirmed Symptoms” search, the coder would check if the post referred to a confirmed symptom of COVID-19 (TP) or whether the matched keywords were mentioned in a different context (irrelevant [I]; also, false positive [FP]). For instance, if a post had been returned by the Boolean search for COVID-19 vaccines, did the post refer to COVID-19 vaccinations? If yes, the post was coded as a TP. If the post in question mentioned COVID-19, but the part of the post mentioning vaccines was about the influenza vaccine, the post would be coded as an FP.

The initial retrieval precision testing showed an average result of 82% for the 35 taxonomy subcategory searches. The retrieval precision rate was calculated as precision = [TP ÷ (TP + FP)] × 100%.

A total of 7 searches returned content below the target minimum retrieval precision rate of 70%, with a range of 42% to 100% (Table 1). To reduce the rate of false positives, the keywords for the 7 searches that performed below the target minimum rate were subsequently reviewed and updated to exclude keywords and phrases returning irrelevant content. On retesting, the average retrieval precision rate for the 35 searches was 87%, with a range of 72% to 100%. The full results of the retrieval precision testing and subsequent retesting can be seen in Table 1.

To spot-check the coding for reliability, we deployed a second reviewer to analyze 10% of the posts (30 per taxonomy category search, 1500 in total). We calculated the Cohen kappa to determine intercoder reliability, which was found to be high (κ=0.81, observed agreement [p_o_]=0.95, expected agreement [p_e_]=0.76).

A further test was performed to assess retrieval recall: whether content of relevance to the research aims failed to be retrieved by the taxonomy searches. To test this, a random sample of 1000 items of content, mentioning COVID-19 (and synonyms) but excluding the taxonomy category keywords (the “not retrieved” sample in Table 2), was human-coded for relevance from a public health perspective. Posts in this sample were determined by the coder to be relevant (R) to the aims of the public health research (false negative [FN]), or irrelevant (I) to the research aims (true negative [TN]). Coding was performed by the same reviewer and was binary; content was irrelevant (I, and therefore also TN) or was relevant (R, and therefore also FN) and deemed to have been missed in taxonomy category searches.

**Table 1:** Results of retrieval precision testing and retesting with a sample size of 300 posts analyzed per subcategory.

Subcategory	Posts retrieved by the taxonomy category search human-coded as true positives and retrieval precision rate, n (%)
The Cause – The Cause	217 (72.3)
The Cause – Further Spread – Stigma	260 (86.7)
The Cause – Further Spread – Immunity	245 (81.7)
The Illness – Confirmed Symptoms	189^a^ (63)/239^b^ (79.7)
The Illness – Other Discussed Symptoms	141^a^ (47)/218^b^ (72.7)
The Illness – Asymptomatic	300 (100)
The Illness – Presymptomatic	300 (100)
The Illness – Means of Transmission	295 (98.3)
The Illness – Protection From Transmission	299 (99.7)
The Illness – Underlying Conditions	238 (79.3)
The Illness – Demographics – Sex	215 (71.7)
The Illness – Demographics – Age	215 (71.7)
The Illness – Vulnerable People	287 (95.7)
The Illness – Vulnerable Communities	269 (89.7)
Treatment – Vaccines	300 (100)
Treatment – Current Treatment	144^a^ (48)/224^b^ (74.7%)
Treatment – Research & Development	290 (96.7)
Treatment – Nonproven Treatment (Nutrition)	245 (81.7)
Treatment – Myths	126^a^ (42)/221^b^ (73.7)
Interventions – Measures in Public Settings	243 (81)
Interventions – Testing	280 (93.3)
Interventions – Supportive Care – Equipment	204^a^ (68)/257^b^ (85.7%)
Interventions – Supportive Care – Health Care	289 (96.3)
Interventions – Personal Measures	298 (99.3)
Interventions – Reduction of Movement	256 (85.3)
Interventions – Protection	276 (92)
Interventions – Technology	278 (92.7)
Interventions – Travel	250 (83.3)
Interventions – Faith	201^a^ (67)/269^b^ (89.7)
Interventions – Unions and Industry	223 (74.3)
Interventions – The Environment	183^a^ (61)/290^b^ (96.7)
Interventions – Inequalities	280 (93.3)
Interventions – Civil Unrest	280 (93.3)
Information – Misinformation	273 (91)
Information – Statistics	244 (81.3)

^a^Indicates a taxonomy subcategory search that performed below minimum requirements and was subsequently updated and retested to yield better performance.

^b^Number and percentage of posts in the sample coded as true positives in the retesting of the taxonomy subcategory search following the update.

**Table 2:** Results of human coding of retrieved and unretrieved samples for calculation of retrieval recall and F-scores.

Sample	Coded relevant		Coded irrelevant		Total coded sample, n
	Samples, n	Description	Samples, n	Description	
Retrieved^a^	875	True positive	125	False positive	1000a
Not retrieved	304	False negative	696	True negative	1000
Total	1179	N/A^b^	821	N/A	2000

^a^The “retrieved” sample size was downweighted to equal the “not retrieved” sample size.

^b^N/A: not applicable.

The results of the coding of the “not retrieved” sample indicated the proportion of TN results as a proportion of the sample; 70% of content was judged not to be relevant to the research aims, and therefore it was deemed correct that this content was not retrieved by our taxonomy. From the data, we also calculated the retrieval recall rate as recall = [TP ÷ (TP + FN)] × 100%.

The overall retrieval recall rate was 74%. This coding process enabled identification of areas where the existing Boolean string could be expanded to include more relevant keywords to retrieve more relevant content, or where the taxonomy could be expanded to include new and emerging issues. From the content that was not retrieved but was judged to be of potential relevance to the research aims (false negatives, FNs), 3 topics were identified that will be added to the taxonomy in a pending update: mutations/variants of the COVID-19 virus; “long covid” (long-term symptoms of COVID-19); and the impact of the pandemic on mental health and well-being.

To validate the coding of the sample of “not retrieved” content for reliability, we deployed a second reviewer to analyze 10% of the posts (100 posts). We calculated the Cohen kappa to determine intercoder reliability, which was found to be high (κ=0.86, p_o_=0.93, p_e_=0.50).

From the results of the coding of the retrieved and unretrieved samples, we calculated an F1 score and an F0.5 score with the following formulas: *F*_1_ = [(2 × precision × recall) ÷ (precision + recall)], and *F*_0.5_ = [(1.25 × precision × recall) ÷ (0.25 × precision + recall)].

The F1 score (harmonic mean of precision and recall) for the searches was 0.80, and the F0.5 score was 0.84. F1 and F0.5 scores range from 0 to 1, with 1 representing perfect performance. A higher F1 or F0.5 score is considered reasonable, with a score closer to 1 indicating stronger performance of a retrieval and classification approach. The inclusion of the F0.5 measure reflects the greater importance of retrieval precision in this study: given the vast number of potentially relevant pieces of content, it is more important to the aims of this project to correctly classify the retrieved posts than to collect every possibly relevant post. Therefore, we consider it a positive result to achieve a higher F0.5 score than F1 score. This is because in this study, it is more important that the results are not impacted by a high number of false positives and that the true positives are classified into the correct subcategory. The retrieval recall testing is also helpful because it enables identification of new or changing pandemic issues, such as new terminology being used that can be added to the taxonomy category search language over time.

### Quantitative Data Analysis

Potential information voids were identified based on 3 parameters within the weekly data set: the volume (ie, how many social media items referred to topic X?), the velocity (ie, the rate of increase of the number of social media items that have engaged with topic X over the course of the past week), and the presence of questions about the topic. The volume was the sum of the online items that mentioned COVID-19 together with a keyword related to each tracked topic. Velocity was determined as the percentage increase of the volume of content items aggregated under each topic from week to week, where velocity = [(current week’s total number of mentions – previous week’s total number of mentions) ÷ (previous week’s total number of mentions)] × 100%.

Starting in late March 2020, weekly global analysis reports were produced that supplied the EPI-WIN team with early warnings of points of concern expressed in public comments by online users [2,4]. By May 4, 2021, the data sample consisted of a sum of 1.02 billion unique social media posts. This was a subset of the larger pool of 1.3 billion total public social media posts in English and French mentioning COVID-19 gathered by the data aggregator. The sample of 1.02 billion posts consisted of approximately 3% of the pool of all public social media posts written in English and French that had been gathered by the data aggregator since March 2020. The data set of total public social media posts gathered by the aggregator was verified through the automated search of mention of the most common words in English and French (eg, *the*, *le*, *and*, *et*).

Each week, social media conversations were segmented based on levels of velocity and quantitatively examined for public engagement (eg, likes, shares, poll votes, reactions), hashtags, and most-used keywords and phrases. From this weekly quantitative data, up to 10 topics with high velocity and/or a large proportion of social media posts expressing a question, and/or with high levels of engagement, were identified as potential priority information voids or sources of confusion or concern.

The identified issues on social media were then further evaluated using engagement data and Google search trends to determine whether a significant number of online users had also been looking for information on these topics to help determine whether the information void was more widespread.

### Qualitative Analysis

Each week, we used the quantitative analysis to identify up to 10 topics reflecting potential information voids and areas of concern. These topics were then examined in more detail via qualitative analysis to understand the context and identify where action may need to be taken in line with a sequential explanatory design approach [30]. The qualitative analysis involved ad hoc human-led review of the key narratives, influencers, and public reactions as reflected in the content.

This analysis prioritized the flagging of widespread confusion or frequently asked questions, the rapid amplification of misinformation, or ad hoc aspects of the conversation that were particularly relevant to public health, such as vaccine questioning ahead of and during a vaccination campaign.

### Reporting

The quantitative data were compiled in a web-based dashboard accessible to the emergency responders in the EPI-WIN team, and insights were discussed with EPI-WIN emergency responders on a weekly basis. The dashboard was updated weekly to allow investigation of short- and long-term trends in volumes, changes in velocity, and the volume of questions for each topic.

Weekly written reports outlined quantitative and qualitative findings about the 5 to 10 topics of concern, included visualizations from the dashboard, and summarized recommendations for action when needed [31].

## Results

Quantitative analysis of the volume changes indicated that the narratives and questions in the online conversations shifted as the pandemic evolved over the course of 2020 and into 2021 (Table 3). Based on the average weekly rises of the topics within each of the 5 taxonomy categories in the yearly quarters between March 23, 2020, and March 31, 2021, it was observed that the second quarter (Q2) and third quarter (Q3) of 2020 were characterized by a steady increase in conversations about “the interventions.” Although discussion of “the illness” decreased in 2020, it surged again in the first quarter (Q1) of 2021. In the fourth quarter (Q4) of 2020, “the treatment” had the highest velocity in digital conversations, while the metaconversation on COVID-19 information experienced the greatest velocity in Q1 of 2021.

**Table 3:** Most discussed topics by month and results of the pivoted data set by month, sorted by volume of social media mentions.

Year and month		Most discussed topic	Volume (millions of social media mentions)
**2020**			
	March	Interventions – Testing	37
	April	Interventions – Testing	18
	May	Interventions – Measures in Public Settings	12
	June	Interventions – Testing	11
	July	Interventions – Testing	14
	August	Interventions – Testing	9
	September	Interventions – Testing	8
	October	Interventions – Testing	17
	November	Interventions – Testing	8
	December	Treatment – Vaccines	15
**2021**			
	January	Treatment – Vaccines	15
	February	Treatment – Vaccines	12
	March	Treatment – Vaccines	15
	April	Treatment – Vaccines	15

At the same time, topics re-emerged periodically in terms of popularity. All 35 categories of topics that were tracked resumed a higher velocity throughout the reporting period for an average of 18 weeks combined (Table 4). The 2 topics that attracted increasing interest most frequently were “myths” and “risk based on age demographics” (rising for 26 and 24 weeks, respectively) followed by “the cause” of the virus and “reduction of movement” (both 23 weeks) “vaccines” and “stigma” (both 22 weeks), and “other discussed symptoms” (21 weeks). Digital conversations on “the cause” of the epidemic, “misinformation” as a phenomenon, and “immunity” had the longest continuous periods of surge in volume of social media posts discussing these topics in the context of the COVID-19 pandemic; the conversation on “the cause” increased in both the first and second half of the analysis period for 7 continuous weeks during the first half of the reporting period, while the metaconversation about misinformation increased for 6 consecutive weeks. Conversations about “immunity” increased for 5 consecutive weeks in June-July 2020 and in November-December 2020.

**Table 4:** Frequency of weekly velocity growth (number of weeks in which a topic experienced positive velocity) and average weekly increase rate (or decrease, when a negative value is returned) by topic.

Topic	Number of weeks in which a topic experienced positive velocity (increase of social media mentions since previous week)	Average weekly increase in number of social media mentions (%)
Treatment – Myths	26	31
The Illness – Demographics – Age	24	8
Interventions – Reduction of Movement	23	14
The Cause – The Cause	23	7
Vaccines	22	7
The Cause – Further Spread: Stigma	22	10
Interventions – Faith	21	3
The Illness – Other Discussed Symptoms	21	52
Treatment – Current Treatment	20	3
Interventions – Travel	20	3
Interventions – The Environment	20	7
The Illness – Confirmed Symptoms	20	1
The Illness – Asymptomatic transmission	19	6
The Illness – Means of Transmission	19	10
Interventions – Measures in Public Settings	18	4
The Cause – Further Spread: Immunity	18	9
Treatment – Nonproven Treatment (Nutrition)	18	4
Information – Statistics and Data	18	4
Interventions – Technology	18	5
Information – Misinformation	17	5
The Illness – Vulnerable Communities	17	4
Interventions – Testing	17	-1
Interventions – Supportive Care – Health Caren	17	3
Interventions – Protection	17	-3
The Illness – Presymptomaticn	16	40
The Illness – Underlying Conditionsn	16	10
Interventions – Supportive Care – Equipment	16	0
The Illness – Protection From Transmission	16	0
Interventions – Personal Measures	15	-3
The Illness – Demographics – Sex	15	8
Treatment – Research & Development	15	8
Interventions – Unions and Industry	15	-1
Interventions – Inequalitiesn	14	-1
The Illness – Vulnerable Peoplen	14	1
Interventions – Civil Unrest	14	32

Analysis of the peaks in discussion of 2 of the leading recurring topics, “risk related to age demographics” and “the cause,” provided insight into how narratives around these topics were fueled by real-life events. The conversation on “risk related to age demographics” increased in velocity 24 times throughout the period studied. A total of 3 million public social media posts engaged with the topic: 64% of these posts were focused on children, whereas 30% focused on older people. The volume of conversation on children and COVID-19 risk increased above the yearly average for 133 days. Speculation about the severity of COVID-19 infection in children was raised consistently throughout the evaluation period, and it represented fertile ground for confusion and potential misinformation. Major triggers included news reports of child deaths (560,000 public posts discussed children and mortality), reports of symptoms observed in children in particular (300,000 public posts discussed children and symptoms), the debate over school reopenings, particularly with regard to transmission (818,000 public posts) and, most recently, COVID-19 immunization (656,000 public posts). In relation to this topic, doubts resurfaced repeatedly about the threat of COVID-19 to children; however, there was a diversity of narrative foci for these doubts, linked to changing events during the pandemic.

By contrast, public discussion on the possible origins of the pandemic (“the cause”) had a persistent narrative throughout the evaluation period. “The cause” of the epidemic was a focus of 3.26 million public social media posts throughout the period monitored. The size of the conversation was most prominent at the beginning of the pandemic and diminished as of June 2020, but with periodically recurring peaks in the number of posts. Conspiracy theories suggesting the artificial origin of the virus as a bioweapon were persistent in online discussion, and prominent influencers operating in the conspiracy theory space were often linked to resurgent peaks in public online discussion. The phrase “biological weapon” was mentioned 326,000 times in the public social media space (cf. 141 million mentions of COVID-19 vaccines in the same period). The rate of mentions decreased by 65% from Q2 to Q3 2020 (as it decreased to 34,000 mentions globally), but it surged to 110,000 in Q4 as the theory regained prominence in the public discourse, in part driven by the release of a preprint paper claiming that the virus was an “unrestricted bioweapon” [31,32]. In Q4 2020, 16% of posts referring to the virus as a bioweapon referenced the authors of that paper. Although the nature of the narrative around COVID-19 as a “bioweapon” was relatively constant, our findings indicate that existing conspiracy theories can be fueled with new details in debates about science [32], underscoring a need to improve science literacy and communication.

## Discussion

### Principal Findings

The insights obtained in this study have afforded public health experts the opportunity for a more rapid and targeted assessment of a subsample of narratives across the English and French languages using public digital sources. These insights can be combined with others to better understand whether and how people are understanding public health and social measures and putting them into practice to protect themselves and their communities.

The application of this taxonomy to successive weekly online social listening analysis has resulted in a better understanding of the evolution and dynamics of high-velocity conversations about COVID-19 worldwide in English and French during the pandemic. The taxonomy also provides a quantifiable approach to support more adaptive and targeted planning and prioritization of health response activities. For example, monitoring and characterizing re-emerging topics can guide re-evaluation and updating of risk communication and community engagement initiatives to improve understandability and resonance, or highlight where adjustments in technical guidance, public health policy, and social measures may be needed. In addition, the fact that narratives discussed online often overlap across different categories reveals the breadth of this taxonomy, and this overlap enables emerging narratives and potential information voids to be picked up through velocity alerts raised in different elements of the taxonomy.

The testing process described in this article forms the basis of the taxonomy review and maintenance process. Updates to the taxonomy are also informed by observations from the weekly analysis and reporting of the data, and public health expert knowledge via WHO, the wider news agenda, and epidemic management context of the pandemic. The taxonomy has been updated twice since its creation in March 2020, with a third update forthcoming in 2021. The aim of the taxonomy updates is to ensure that important new and emerging topics are captured as the pandemic evolves (as in the examples of variants/mutations and “long covid” above) and that the taxonomy includes the latest language and terms being used by the public [29]. For example, as the pandemic progressed, members of the public increasingly dropped the use of formal terms, such as referring to the virus as “Covid” rather than “COVID-19”; therefore, the taxonomy keywords were expanded to reflect this change. When the taxonomy was updated and validated, the database was also updated back to the start date of the research to ensure consistency in the analysis data set and to allow for analysis of long-term trends.

There is added value in using a common social listening taxonomy for integration of insights from a variety of data sources and research methods in online and offline communities. This can provide a more systematic way to integrate analysis of different data sources and facilitate complementarity of digital social listening data with other data such as knowledge, attitudes, and practices research to help uncover drivers of online discussion, and to support social listening in vulnerable or more marginalized communities, including those with limited access to online platforms.

A challenge of this analysis approach is the need for human analysts to continuously monitor and evolve the taxonomy in line with the developing narratives and emerging topics as well as the changing language used in discussions of the COVID-19 pandemic. Ideally, taxonomies would be tested, reviewed, and updated frequently, particularly when a new stage of the pandemic begins (eg, when the vaccine rollout started), as such events in the pandemic timeline can generate new topics of discussion and new terminology (eg, “Covid passports”). However, the benefit of more frequent updates is balanced by the need for comparability of data across time as well as by the fact that this analytical method needs to be rapidly reproducible, including in more resource-constrained environments, to have real, practical use week-on-week during the pandemic to inform the immediate needs of the health authority response, including risk communication and community engagement in any country context.

To help identify actionable insights, the weekly analysis was focused less on exact counts of mentions and more on relative changes, narratives, and topic signals to evaluate and contextualize infodemic signals. When rapidly identifying up to 10 information voids in large weekly data sets, absolute precision was less important than the early detection of an actionable signal to help trigger a timely response. For example, if there was a sudden rise in online narratives expressing concern over a treatment, coupled with other information available from the emergency response, the exact number of mentions was less important than signal detection, analysis, and recommendations for possible action. Despite this, more research is needed to refine and streamline the process for rapidly updating and publishing such taxonomies, especially in protracted epidemics, where shifts in concerns and conversations are bound to occur.

A key takeaway from the analysis that can be applied during the current pandemic is the frequent recurrence of topics of concern and its implications for communication. Public health authorities, governments, and nongovernmental organizations must be prepared to communicate repeatedly on the same issue, adapting frames, approaches, and content as public perceptions of issues and topics shift. Our analysis shows that areas of concern wax and wane, with confusion disappearing and re-emerging as new information comes to light or new events occur. Monitoring the changing narratives on a weekly basis and over time using a taxonomy, such as the one used in this study, can enable health authorities to assess longer-term trends and to be more nimble in adapting approaches to respond effectively to topics of concern and to counter misinformation. Further research can help to adapt these digital social listening approaches to provide metrics for evaluation of infodemic management interventions.

The taxonomy has been adapted, translated, and applied in a number of country-level studies in Mali, the Philippines, and Malaysia [33-35]. Applying the approach at the country level included the localization of keywords and their validation. Once this work was completed, the taxonomy and methodological approach proved to be a useful tool for generating insights into narratives in public discourse and potential information voids at the national level. Furthermore, the research framework is now being applied in Canada by the National Institute of Public Health of Québec as an input into the public health response and risk communication in that province, showing that the taxonomy is also applicable at the subnational level [36]; Institut national de santé publique du Québec [forthcoming].

A pilot project by WHO EPI-WIN and research partners, Early Artificial intelligence–supported Response with Social Listening (EARS) [37], also built on the taxonomy from this research and applied it to an automated classification of content and analysis of publicly shared opinions and concerns in 20 countries. The EARS project is enabling both country-level analysis and cross-country comparisons of themes in online conversations, although obtaining in-depth contextual insights still requires human-led analysis of potential information voids and sources of confusion. Therefore, more investment in analytical capacities in social listening at the country level is needed to provide more contextual analysis, interpretation of infodemic insights, and formulation of recommendations for action, as well as to build capacities for using social listening for health response evaluation and adaptation.

There is an opportunity to apply the taxonomy and methodology described in this paper to detect information voids during future, as yet unknown, pandemics and other public health crises. The 5 top-level categories and some of the 35 sub-categories are relevant to social listening in any outbreak but would need to be adapted to the type of pathogen. If, for example, the HIV/AIDS epidemic had started in the digital, connected world of 2020 rather than in the 1980s, the online social listening taxonomy structure would have needed some adjustment to filter and segment public discourse related to the epidemic and identify information voids. For example, a “Demographics – Men Who Have Sex With Men” topic could be added under the category “the illness” to better hear questions and concerns from this particular demographic group. This approach could also include adjustments to subcategories under “the intervention” to remove irrelevant subcategories of “Reduction of Movement” and “Unions and Industry.” After such a taxonomy review and adjustment, the keywords used to capture content related to each category and subcategory would also need to be systematically reviewed to ensure they were appropriate to the narratives in relation to specific illness in question. For example, terms relating to injected drug use, sex between men, sex between a man and woman, and mother-to-child transmission could be added under “The Illness – Modes of Transmission”. Having a taxonomy structure and methodology already in place as a starting point would enable faster deployment of digital social listening activities in a future outbreak.

### Limitations

Interpretation of the analysis must account for the limitations of the data sources included in the content aggregator. During health emergencies, health authorities require surge support in social listening, response, and evaluation functions. Analysis services from a central analytics unit or from commercial or academic institutions need to be set up quickly to use a systematic approach to detect and understand people’s changing concerns, questions, and possible areas of confusion shared publicly online. The overhead in management of data from open sources can be high, and in settings where the social listening analytics capacity is not yet in place for routine analysis, content aggregators can be used to rapidly set up an analysis workflow. The media content aggregation platform used for this study offers firehose access to Twitter, ensuring a complete set of data for analysis, subject to privacy limitations. Other sources in the platform are either sampled from or limited to public posts only [38]. This is a limitation that applies to most analytics of this type, as Facebook and other social media platforms set limitations on the data they make available due to their privacy policies and commercial interests. As a result, there is an overrepresentation of Twitter content in this analysis [39,40]. The use of private data aggregators may lead to the use of unconventional, uncontrolled samples whose breadth and comprehensiveness are constrained by practical and legal limitations. Other methods would be required to characterize conversations in hidden online communities, closed groups, and closed messaging apps, and thorough consideration of the ethics of social listening would be warranted in such contexts.

This research is global and is limited to two major languages (English and French). As a result, only major online narrative themes and information voids were identified, and the resulting interpretations may not be representative of trends and patterns that could be observed in digital communities for other languages. Moreover, in a global weekly analysis, smaller or more localized conversations may go undetected. One of the aims of this work is to apply and advance the methods to develop taxonomies that can be rapidly applied to any linguistic context for different geographies and public health events.

It has also been observed that the global English-language data set is prone to overrepresent the voice of social media in geographic regions or communities that are more digitally active than others. A key challenge in this study was the digital amplification of discourse pertaining to US politics, the elections, and the digital prominence of US civil society thereof [41]. In such situations, exclusion keywords may be used to exclude major events or large-scale media coverage from analysis so that they do not mask citizens’ publicly shared narratives that are more relevant for public health authorities. This can also be addressed when presenting the analysis results. For example, the weekly reports presented analysis of the narratives from the United States and the United Kingdom separately from the analysis of data from other countries where English was the language of online conversation. This helped to uncover previously undetected narratives outside the United States and the United Kingdom. Future research is needed to assess how results may vary in different linguistic communities and to evaluate the effects of geographies that may be superinfluencers of global discourse.

Another limitation of this research is the start date of the project, March 23, 2020, which is several weeks after COVID-19 was declared a public health emergency of international concern; however, data prior to this date (back to January 2020) have been retrieved and stored for future analysis, ensuring that it is possible to analyze a longer timeline.

Adaptation and application of the taxonomy structure in future outbreaks must also take into account validation of information retrieval and recall. The test scores referenced in the taxonomy testing and validation section should be taken as estimates of the accuracy of the retrieval process by the taxonomy category searches, and function most effectively as a tool for identifying areas for improvement. A key limitation of the test results is that human coders can make errors [29]. The human coders involved in the testing and validation were highly experienced in coding and highly familiar with the topic in question, which can help minimize the incidence of coding errors. Future applications of this validation approach could also deploy more coders in an effort to remove potential bias introduced by reliance on a small number of coders.

### Conclusions

This research focuses on the identification of potential information voids and sources of confusion in online social conversations to provide actionable insights for risk communication and community engagement and other health response activities. While it can provide insight into the opinions expressed online, integration with other analyses, including from listening to offline communities is needed. Applying this methodology globally has provided the added and needed insight, inspiring new ways of thinking and use of information in support of risk communication during health emergencies. Much of the value of the taxonomy we developed is in the capacity to rapidly deploy and provide ongoing insights about information voids during an outbreak, which then allows a health authority to take evidence-informed action and course-correct risk communication during an epidemic. The application of the taxonomy and methodology for social listening at regional, country, and subnational levels in the COVID-19 pandemic—which is already being tested—offers possibilities for more actionable insights that must increasingly support a localized response. Moreover, this method offers an approach for monitoring of concerns, questions, and information voids in future outbreaks, enabling a faster response by the health authorities in affected countries during the next acute health event.

### Data Availability

The listing of keywords and search terms per taxonomy subcategory is available upon request by contacting enquiry@mediameasurement.com.

## Abbreviations

EARS: Early Artificial intelligence–supported Response with Social Listening
EPI-WIN: World Health Organization Information Network for Epidemics
FN: false negative
FP: false positive
pe: expected agreement
po: observed agreement
Q1: first quarter
Q2: second quarter
Q3: third quarter
Q4: fourth quarter
TN: true negative
TP: true positive
WHO: World Health Organization
